# Supplementary material for: Nano-scale solution of the Poisson-Nernst-Planck (PNP) equations in a fraction of two neighboring cells reveals the magnitude of intercellular electrochemical waves
Source: PLoS Comput Biol. 2023 Feb 15;19(2):e1010895. doi: 10.1371/journal.pcbi.1010895 (PMC9974139; doi:10.1371/journal.pcbi.1010895)
Supplement: Attachment — Submitted filename: Response_to_reviewers.pdf [file pcbi.1010895.s003.pdf]

# Response to reviewers' comments

We are grateful for the careful reviews undertaken by both reviewers and for the editorial comment.

## Editorial comment:

This paper makes important advances from a numerical methods perspective, but very little attention is made to how these predictions may be validated experimentally or even used to guide future experiments. The discussion should address this as appropriate.

**Authors' reply:**
Since the space between neighboring cardiomyocytes is very narrow, it is difficult to measure the consequences of ephaptic coupling directly. In the revised version, we have added the following text concerning this issue:

«Providing direct evidence of ephaptic coupling is difficult because it is technically challenging to measure extracellular potentials in intercalated disc clefts that are only a few tens of nanometers wide. Thus, for the moment, experiments provide only indirect evidence. For example, in [21] the authors examined in voltage clamp experiments how restricting the extracellular space near a cell expressing voltage-gated $Na^+$ modifies the resulting $Na^+$ current. It was found that at step potentials just above the excitation threshold, the $Na^+$ current was increased, whereas at step potentials far above threshold, the $Na^+$ current was decreased. Both phenomena are compatible with ephaptic effects and were in line with modeling predictions.

An interesting method to measure extracellular potentials, called "diamond voltage imaging", has been recently developed, see [52]. This technology allows measuring extracellular potentials in an electrolyte solution at high spatial resolution, and it may thus open perspectives to provide direct extracellular measurements in narrow extracellular domains. Nanowires that could be inserted into narrow clefts may represent another option.

Chemical or optogenetic reporters of electrical potential or even of ion concentrations that could be directly targeted to the intercalated disc may represent a further interesting approach in the future. However, at the present time, optogenetic reporters exhibit kinetics that are still too slow to permit the detection of rapid potential or ion concentration changes, which, as indicated by our present study, may occur already at the nano- and microsecond scales. Chemical probes are probably more appropriate, but the development of such probes will undeniably by a major challenge.
Hence, for the time being, modeling and simulations represent a useful and important approach to gain insight into the behavior of ion channels, electric potentials and ion concentrations in biological nanodomains.»

## Reviewer 1:

1. First, in Figure 14, can you provide the corresponding values in the right panel plots for the right cell? In particular phi_e_min would be relevant to understand how hyperpolarized the extracellular potential is adjacent to the $Na^+$ channels on the right cell membrane.

   This detail in general is relevant to my largest question. Is the hyperpolarization in the extracellular space adjacent to the right cell sufficient to activate $Na^+$ channels on the right cell membrane in physiological conditions? The trend that phi_e_min decreases (becomes more negative) as the membrane area increase suggests that the hyperpolarization would be larger in a realistic area (as for the upstroke duration), but the trend is not as clearly linear as the upstroke duration. Can the simplified model be applied to estimate the phi_min_e for a larger area? If not, can the authors comment on this more broadly - even if it is speculation - as to their expectations?

   **Authors' reply:**
   We have now included the corresponding values for the right cell in Figure 14.

   In these plots we see that like for the left cell, phi_e_min becomes slightly more negative as the membrane area is increased. However, as the reviewer points out, the trend is not as clearly linear as for the upstroke duration. When the membrane area is increased from 300 nm x 300 nm = 0.09 um^2 to 2000 nm x 2000 nm = 4 um^2, i.e,. by a factor of about 45, the upstroke duration is increased by a factor of about 50 (from 255 ns to 13,530 ns), whereas the phi_e_min is only decreased by a factor of about 1.5 (from -5.2 mV to -7.6 mV).

   The simplified model only includes the membrane potential, and not the intracellular and extracellular potentials separately, so it cannot be used to estimate phi_min_e for a larger area. We therefore find it hard to speculate exactly how phi_e_min outside of the right cell would change as the membrane area is increased to a more physiologically realistic setting, but we have included the following comment in the manuscript (Section 3.2.5):

   "We also note that the results in Figures 13 and 14 indicate that the magnitude of the change in the extracellular potential and the ion concentrations increases as the membrane area is increased. From these observations, we expect that the changes in potential and concentrations in a simulation with a more realistic membrane area would be at least as large as those observed in our small simulations with a membrane area of 300 nm × 300 nm (see Figures 9–12), and possibly even larger. Nevertheless, this estimate is associated with a level of uncertainty, since the membrane areas currently investigated are considerably smaller than the membrane area needed for realistic durations of the dynamics and because the simplified model is not suitable for estimating changes in the extracellular potential or ion concentrations following from an adjusted membrane area."

2. Regarding the description of the charge density profile near the $K^+$ channel in Figure 7 (bottom row), even after reading the description in the Supplement, I was a bit confused, and I think the description could be a bit more clear.

   The authors state that the complex charge density profile arises due to small deviations from the linear profile used as $[K^+]$ in the initial conditions, but would it be more accurate to describe the complex profile as due to the channel $[K^+]$'s deviation from a linear profile at steady-state (not initial conditions)?

As I understand, the assumption of a linear profile in [$K^+$] for initial conditions also results in a linear profile in rho_0. Since the steady-state $K^+$ in the channel deviates from the rho_0 profile, the complex rho profile emerges.

**Authors' reply:**
We agree that the description given here was unclear. Our comment about the charge density profile was meant to say that the profile was a consequence of the deviations **at steady state** from the purely linear profile used as initial conditions, like the reviewer suggests. We have tried to make this clearer in the manuscript by rewriting the comment to:

"The profile of rho in the $K^+$ channel cluster is a consequence of small deviations at steady-state from the purely linear profile used for rho0 and initial condition for the $K^+$ concentration in the cluster and is discussed further in the Supplementary Information."

3. Line 151: The authors' state "After the simulation in Part 1 seems to have reached a steady state, ..." please be more specific as to what was considered to determine if the Part 1 system had reached steady state.

   **Authors' reply:**
   We have now included the following specification in Section 2.2:

   "We run this simulation until we seem to have reached a steady-state solution for open $K^+$ channels. More specifically, we run the simulation until the change in the computed transmembrane potential (see Section 2.4.2) is reduced by a factor of at least 500 compared to the maximal change in the transmembrane potential occurring in the beginning of the simulation."

4. In Figure 9, please comment and specify the value of the time associated with each snapshot. This would enable commenting on how the number of channels and extracellular space width impact the time that the greatest cleft polarization occurs, which in turn may relate to the efficacy of ephaptic transmission.

   **Authors' reply:**
   We have now included the time associated with each snapshot in Figures 9-11, as well as in the corresponding supplementary figures.

5. In Figure 15, top row, was the voltage-dependence of $Na^+$ channel closure incorporated into the simplified model? I would expect that the voltage traces would return back to $E_K$, when the $Na^+$ channels are closed but instead it reaches a plateau.

   **Authors' reply:**
   Thank you for pointing this out. The voltage-dependence of $Na^+$ channel closure was not incorporated into the simplified model, but the plots in the lower panel of Figure 15 seemed to suggest it was. We have now updated the plots and include the following comment in the manuscript (Section 3.2.4) and in the caption of Figure 15:

“Note that since we assume that the open probability of the $Na^+$ channels is 1 ($o_{Na}$ = 1) in the simplified model, whereas the $Na^+$ channels close when v reaches a value of 30 mV in the PNP simulations, we only use the simplified model to approximate the PNP model during the upstroke of the action potential, before the $Na^+$ channels close.”

- Minor: I leave this to the authors' discretion, but the use of 'left' and 'right' cell seems a bit arbitrary, and something such as pre- and post-junctional or 'upstream' or 'downstream' could be more appropriate.

  **Authors’ reply:**
  We agree and we have updated the manuscript using the terms “pre- and post-junctional cells”.

- Minor: Provide a definition and numerical value for beta_$Na^+$ in Eqn. 20.

  **Authors’ reply:**
  We have now added the following comment after Eqn. 20:

  “The parameter $\beta_{Na+}$ is defined in (5) and has a value of 0.037 $mV^{-1}$ for the parameter values specified in Table 3.”

- Minor: Can you produce a summary plot comparable to Figure 12 for the extracellular ion concentrations as well? This concise summary would be helpful.

  **Authors’ reply:**
  We have now included the extracellular concentrations in addition to the extracellular potential in Figure 12. In addition, we have included the following comment about the results in the manuscript text (Section 3.2.2):

  “Similarly, the ion concentration changes outside of the open $Na^+$ channel cluster of the pre-junctional cell generally seem to increase when the cleft width is decreased or the number of $Na^+$ channels is increased. However, there are some deviations from this general tendency. In particular, the ion concentration changes seem to be similar or even smaller when the number of channels is increased from 196 to 900. In addition, the ion concentration changes outside of the pre-junctional cell seem to be very similar for Le = 10 nm and Le = 30 nm. Outside of the post-junctional cell, the ion concentration changes are smaller than outside of the pre-junctional cell, and, as expected, the difference between the deviations in ion concentrations outside of the two cells seem to become larger as the cleft width is increased.”

- Minor: Typo on line 481 "maxmimum"

  **Authors’ reply:**
  This typo has now been corrected.

## Reviewer 2:

Minor comments and stylistic suggestions:
- line 40: “perturbing the dynamics” --> “perturbing its dynamics”
- line 48: “the upstroke takes” --> “the upstroke of the AP takes”
- line 50: “to represent that type of areas in” --> “to represent that area scale in”

**Authors’ reply:**
We have incorporated the suggested changes in the updated manuscript.

- Table 1: The chosen initial value for intracellular Na+, 12 mM, is rather high. At least to me, it sounds like a value typical for rodent cardiomyocytes, or maybe failing human cardiomyocytes. In healthy human cardiomyocytes, something like 8 mM would be more realistic. This is just a general note. I do realise that the authors do not explicitly state that their modelling effort is in the context of human cardiomyocytes, even though refs 41 and 42 indicate towards that. Furthermore, I would not expect a change from 12 to 8 mM to affect the findings in any relevant way.

**Authors’ reply:**
We have now performed some simulations where the intracellular $Na^+$ concentration is set to 8 mM instead of 12 mM, and the results seem to be very similar for the two cases. We have included figures comparing the results in the supplementary information (Section S7) and included the following comment in the main manuscript (Section 2.1.1):

“In the supplementary information, we also show the results of simulations with a different choice of initial conditions for the intracellular concentrations. More specifically, the intracellular $Na^+$ concentration in cardiomyocytes is known to vary between species, with a concentration of about 4–8 mM for most mammalian species, but 10–15 mM in rat and mouse [30, 31, 32, 33]. Thus, the default value of the intracellular $Na^+$ concentration reported in Table 1 (12 mM) is mostly representative of rat or mouse cardiomyocytes, and a value of about 8 mM would be more realistic for healthy human cardiomyocytes [31]. In the supplementary information, we compare the results of simulations using either 12 mM or 8 mM as initial conditions for the intracellular $Na^+$ concentration. In addition, the intracellular $Cl^-$ concentration is set to either 137.0002 mM or 133.0002 mM, respectively, in order to maintain electroneutrality at $t = 0$. In the supplementary figures, we observe that the two choices of initial conditions give very similar results.”

- line 193: “The concentrations are in this area fixed at” --> “The concentrations in this area are fixed at”
- line 343: “reach the the neighboring” --> “reach the neighboring”
- line 482: “extremely fines meshes” --> “extremely fine meshes”

**Authors’ reply:**
We have incorporated the suggested changes in the updated manuscript.

- Discussion: To improve readability, I suggest that the authors consider using subheadings: 4.1 Formation of Debye layers, 4.2) Extracellular ionic dynamics, 4.3 Impact of $Na^+$ channel cluster and cleft width, and 4.4 Limitations and perspectives.

**Authors’ reply:**
We agree and have now updated the subheadings as suggested.
